# Knowledge, Confidence, and Comfort Regarding Sickle Cell Disease Among Medical Students: A Pilot Study in Two Universities

**DOI:** 10.3390/healthcare13151909

**Published:** 2025-08-05

**Authors:** Christina M. Abrams, DeAsia Witherspoon, Everette Keller, Andrew J. Picca, Maria Boucher

**Affiliations:** 1 Division of Hematology/Oncology, Department of Pediatrics, Medical University of South Carolina, Charleston, SC 29425, USA; 2 College of Medicine, Medical University of South Carolina, Charleston, SC 29425, USA; withedea@musc.edu; 3 Department of Public Health Sciences, College of Medicine, Medical University of South Carolina, Charleston, SC 29425, USA; kellerev@musc.edu; 4 Division of Hematology/Oncology, Department of Pediatrics, University of North Carolina, Chapel Hill, NC 27599, USA; maria_boucher@med.unc.edu

**Keywords:** sickle cell disease, medical education, healthcare disparities, clinical training, medical student

## Abstract

Background: Quality care of individuals with sickle cell disease (SCD) is dependent upon education of the providers on their care team. Previous studies demonstrate lack of resident and provider comfort regarding care of patients with SCD, yet none have assessed these in medical students. Objective: This study aims to evaluate the adequacy of the research instrument for measuring medical students’ knowledge, confidence, and comfort regarding SCD and related complications prior to wider distribution. Methods: A self-assessment survey was distributed to medical students at two universities to evaluate their knowledge, confidence, and comfort in general SCD topics, in all clinical settings, and regarding common complications. Results: Of the 98 responses, knowledge (*p* < 0.001) and confidence (*p* = 0.02) were significantly different between topics, including epidemiology and genetics, pathophysiology, and treatment options. For “treatment options”, there were significant differences in knowledge (*p* = 0.02) and confidence (*p* = 0.02) between medical students at different levels of training. Students felt least knowledgeable and least comfortable with care of pregnant women and most knowledgeable and most comfortable with acute pain management. Caring for patients with specific SCD-related conditions increased knowledge and comfort across all domains. Conclusions: This instrument was adequate for measuring knowledge, confidence, and comfort in caring for those with SCD across all clinical settings. We identified a lack of knowledge, confidence, and comfort regarding treatment for those with SCD starting early in medical careers, which improves after caring for patients with various complications. Thus, educating and providing SCD patient experiences is crucial for medical student management confidence related to SCD.

## 1. Introduction

Sickle cell disease (SCD) is an inherited hemoglobinopathy affecting approximately 100,000 Americans, as well as an estimated 515,000 babies born worldwide from 2000 to 2021 [1,2]. Currently, more than 90% of people in the United States with SCD are non-Hispanic Black or African American, and 3–9% are Hispanic or Latino [2]. Patients with SCD are prone to acute and chronic complications such as stroke, retinopathy, bacteremia, and severe episodes of pain [3]. Patients also experience serious and organ-specific complications such as acute chest syndrome, avascular necrosis, pulmonary hypertension, and splenic sequestration [3]. These complications of SCD have long-lasting effects on patients’ health and often cause severe, and potentially life-threatening, consequences [3].

Due to the complexity of the disease, patients with SCD require medical attention across the medical system through various specialties [4,5,6]. Encountering patients with SCD is common in medical training and in medical practice, thus justifying the need for all care team members to be knowledgeable about SCD, its varied presentations, and many complications. Gaps in knowledge and comfort in providing care for those living with SCD are highlighted across the literature. Studies emphasize this concern for medical residents demonstrating challenges in the complex nature of these patients, discomfort in ability to provide care, and a desire for better education [7,8,9,10]. Those in medical fellowship have also reported discomfort in caring for these individuals, challenges in managing their complications, and again a desire for SCD specific education [8,11]. It is hypothesized that this deficiency begins in medical school training. However, few studies address this. In addition, there are no currently validated methods to assess these deficiencies.

These concerns continue throughout practice. Primary care providers have reported lack of comfort providing ambulatory care to patients with SCD, even when care is unrelated to their underlying SCD [12]. This suggests comfort was possibly related to insufficient experience in caring for these individuals, and this may result in patients not receiving optimal care [12]. Various models have been trialed to support providers and patients, including tele-mentoring [13], specialized medical homes, and hub and spoke model, which demonstrate variable success [14]. These models depend upon primary care providers as part of the care team for patients [14]. With an ever-shrinking workforce of adult and pediatric hematologists, more general practitioners need to have comfort caring for patients with SCD [15,16]. Studies have reported use of emergency services related to lack of primary care providers specializing in SCD [17]. Similarly, high rates of readmission are related to those without a primary care provider [17,18]. Globally, these problems are echoed across healthcare professionals as well [19]. It is, therefore, vital for all physicians, regardless of specialty, to be confident and comfortable in their ability to care for and treat patients with SCD.

While studies have explored primary care providers’, pediatric residents’, and hematology/oncology fellows’ experience caring for those with SCD [7,11,20,21,22], our pilot study aims to evaluate our survey to assess medical students’ knowledge, confidence, and comfort regarding SCD and treatment of related complications. Additionally, we hypothesize comfort, confidence, and knowledge related to SCD would be low; however, this would increase as exposure to patients was provided.

## 2. Materials and Methods

### 2.1. Development and Distribution

This pilot study was conducted at the Medical University of South Carolina (MUSC) and the University of North Carolina Chapel Hill (UNC-CH) and included students classified as second- through fourth-year medical students (MS2-MS4) at their respective institutions. Both institutions are large, tertiary healthcare systems who each care for approximately 900 patients with SCD per year and are home to comprehensive sickle cell centers. The institutional review board at each institution approved this study (MUSC #Pro00135719 and UNC-CH #24-0570).

Data were collected using an online, self-administered, voluntary survey that was created and distributed using the Research Electronic Data Capture (REDCap) software v13.4.13 [23]. This survey, the Sickle Cell Disease Knowledge and Comfort Survey (SCD-KCS), was newly developed for this specific purpose as no previously validated instruments were found throughout the literature. The SCD-KSC was created and designed to understand medical students’ knowledge, comfort, and confidence in caring for those with SCD. There are no validated surveys in the literature that serve this purpose at any level of training. As such, we selected topics that relate to the most common complications (i.e., acute vaso-occlusive pain, acute chest syndrome, acute stroke) and states (i.e., pregnancy and outpatient management). The SCD-KCS was created with input from hematology providers and a medical student on the research team for the study. Initial development of the SCD-KCS was by first author (CMA) and second author (DW) after review of literature and existing institutional resources. The SCD-KCS was subsequently reviewed and edited by senior author (MB). The final version was approved by all members of the team prior to distribution. Within the distribution information for the SCD-KCS, statements regarding informed consent, privacy, and rights of students were included (Supplemental Material S1). By filling out the SCD-KCS, students were consenting to participate in the project. The SCD-KCS was disseminated to each medical school class’s electronic listserv via direct link. The population surveyed was a convenience sample population. A convenience sample was felt to be appropriate due to size and expertise available at these institutions, as well as time and budget limitations. The SCD-KCS contained three sections: (1) demographic information, (2) a self-assessment of knowledge, confidence, and comfort regarding different aspects of SCD (genetics and epidemiology, pathophysiology and treatment options) and of several SCD related complications (i.e., acute pain, acute stroke, and acute chest syndrome) and other clinical care (i.e., pregnancy management and routine care), and (3) a 10-question objective assessment of knowledge (Supplemental Material S2). Within the SCD-KCS, knowledge and confidence related to clinical topics, while knowledge and comfort related to treatment options. Some answer choices were not included in analysis due to lack of coordinating responses across all elements. Students were asked to rank each question on a Likert scale of 1 to 5, 1 having no knowledge (confidence or comfort) and 5 being extremely knowledgeable (completely confident or comfortable). Students were allotted a 2-week time limit to complete the survey at their convenience. No incentives were offered for survey completion; reminders were sent to students at MUSC 1 week and 1 day before the deadline. For UNC-CH students, a reminder was sent 1 week prior to the deadline.

The pilot survey was sent to 1203 medical students in March 2024. One hundred and twenty-two individuals started the SCD-KCS, for a total response rate of 10.0%. Individuals who did not complete the SCD-KCS were removed (19 individuals), and any individual who did not indicate their institution was also removed (4 additional individuals). One additional person was removed since they did not indicate which year they were in their medical school training, leaving 98 individuals for analysis.

### 2.2. Statistical Analysis

Demographics were summarized for the MUSC and UNC-CH groups of study students. Proportions were calculated for each categorical subset in the respective university. Since the SCD-KCS focused on assessing students’ knowledge, confidence, and comfort regarding SCD and treatment of various related complications, we examined (1) overall differences in knowledge and comfort of three general aspects of SCD (epidemiology and genetics, pathophysiology, and treatment options), (2) differences in knowledge and comfort of the above between class year and institution, (3) overall differences between knowledge and comfort with treatments of SCD specific complications, (4) differences in knowledge and comfort of the above between institutions, and (5) differences in knowledge and comfort in treatments based on experience in caring for a patient with SCD. Overall outcomes for examining the disparities between the knowledge or comfort were analyzed using Friedman’s test for ordinal measures, a non-parametric alternative to repeated measures ANOVA. Comparisons between class year, institutions, and previous treatment experience were analyzed using the Kruskal-Wallis test with repeated comparisons of the same groups across different outcomes being adjusted for multiple testing using a Bonferroni correction. The Friedman’s and Kruskal-Wallis tests are appropriate for detecting differences in the distributions of the three aspects of SCD since the aspects are ordinal and would violate the normality assumption of ANOVA. These tests evaluate whether the distributions of ranks differ significantly either across repeated conditions or across groups, respectively. *p*-values < 0.05 were considered statistically significant. All statistical analysis was performed using R version 4.1.3.26 [24].

### 2.3. Post-Hoc Power Assessment

A post-hoc power analysis was conducted where the observed sample size, *n* = 98 subjects, provided 80% power to detect small differences (e.g., Kendall’s W effect sizes equivalent to 0.04) between SCD aspects, assuming a two-sided hypothesis testing and an alpha level of 0.05. The Kendall’s W of 0.04, which reflects a small effect size, is equivalent to the Friedman χ^2^ = 7.84. Due to the estimated Friedman χ^2^ = 35.2, the study was sufficiently powered to detect small differences in the SCD aspects.

## 3. Results

### 3.1. Demographic Characteristics

The demographic characteristics of the pilot study cohort are summarized in Table 1. Of the 98 students, 61 (62%) were from MUSC and 37 (38%) were from UNC-CH. Overall, there was equal distribution of the three medical school classes, though the class proportions were significantly different (*p* < 0.001), with MUSC having more individuals in the 2nd-year class (51% v 3%) relative to UNC-CH and UNC-CH having more individuals in the 4^th^-year (68%% v 15%) class compared to MUSC. Additionally, MUSC students had significantly greater exposure to SCD from trainings or workshops (33% v 8%, *p* = 0.006) relative to UNC-CH students. In general, the groups of students from the two institutions were comparable with respect to demographics (gender, race, and ethnicity), prior SCD experience (personal, education exposure, media, and other), and career goals. Five (5%) individuals had personal experience when it related to SCD, and most individuals (99%) received information from education exposure, readings, and lectures. About half of the individuals (49.5%) reported having goals of going into primary care.

### 3.2. Overall Difference in Knowledge and Confidence

**Figure 1:**
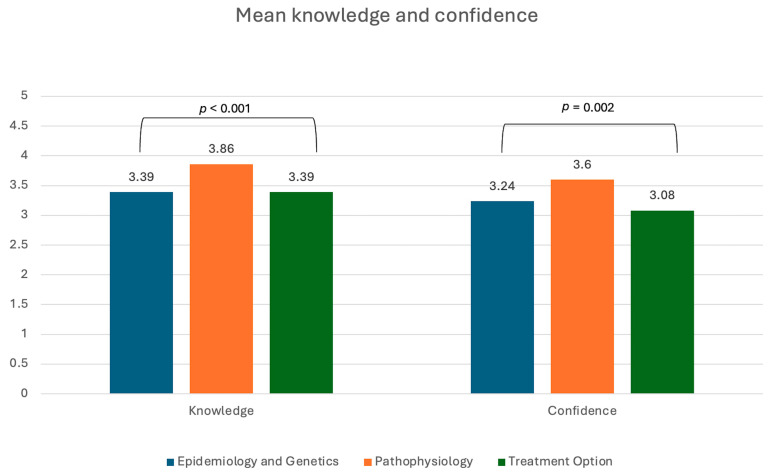
Overall self-reported knowledge and confidence in three aspects of sickle cell related care using Likert Scale values of 1 to 5 (1 = least knowledgeable (or confident) and 5 = most knowledgeable (or confident)). **Note:** *p*-value was determined by Friedman’s Test for Ordinal Measures, a non-parametric method for comparing more than two related groups based on ordinal measures.

Differences in self-reported knowledge of three aspects of SCD related care are represented in Figure 1 below. There was a significant difference (*p* < 0.001) observed in self reported knowledge, with students indicating higher knowledge of pathophysiology on the Likert scale (mean 3.85 ± 0.70; 95% CI: 3.71, 3.99) relative to the categories of epidemiology and genetics (mean 3.39 ± 0.71; 95% CI: 3.25, 3.53) and treatment (mean 3.39 ± 0.83; 95% CI: 3.23, 3.55). There was also a significant difference (*p* = 0.002) observed in self-reported confidence, with students indicating lower confidence of treatment options (mean 3.08 ± 0.95; 95% CI: 2.89, 3.27) relative to the categories of epidemiology and genetics (mean 3.24 ± 0.94; 95% CI: 3.05, 3.43) and pathophysiology (mean 3.60 ± 0.80; 95% CI: 3.44, 3.76).

### 3.3. Self-Reported Knowledge and Confidence Based on Year of Medical School Training

**Figure 2:**
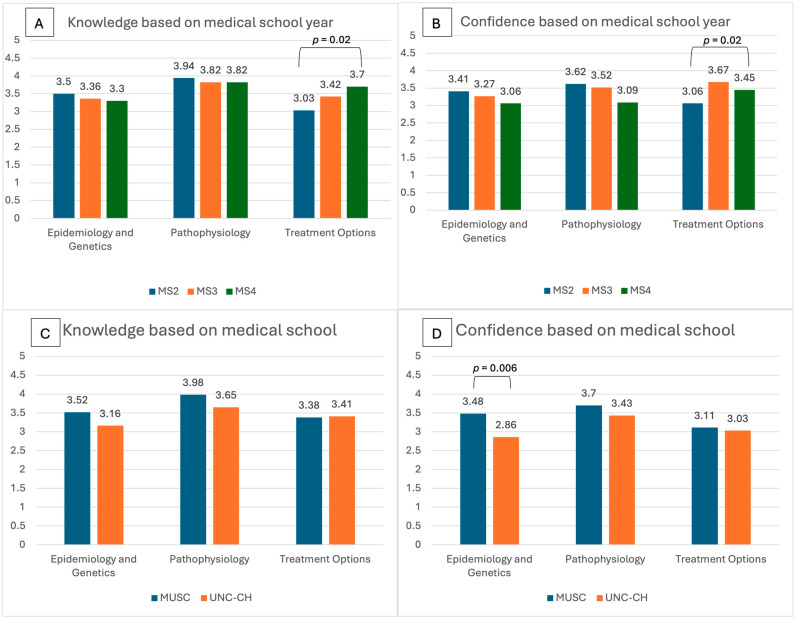
Self-reported knowledge and confidence in three aspects of sickle cell related care from results of SCD-KCS using Likert Scale values of 1 to 5 (1 = least knowledgeable (or confident) and 5 = most knowledgeable (or confident)). Panel A and B compare mean Likert Scale values across the year of medical school training related to knowledge (**A**) and confidence (**B**). Panel C and D compare mean Likert Scale values between institutions related to knowledge (**C**) and confidence (**D**). **Note:** MS = medical student, MUSC = Medical University of South Carolina, UNCH-CH = University of North Carolina, Chapel Hill. *p*-value was determined by the Kruskal-Wallis Test, a non-parametric method for comparing the medians of multiple groups with repeated comparisons adjusted for multiple testing using the Bonferroni correction.

Differences in self-reported knowledge of three aspects of SCD related care are represented in Figure 2 for class year and institution. In Panel A, adjusting for multiple comparisons, there was a significant difference in self-reported knowledge of treatment options (*p* = 0.02) between class years. Knowledge of treatment options increased from 2nd year (mean 3.03 ± 0.93; 95% CI: 2.71, 3.35) to 3rd year (mean 3.42 ± 0.79; 95% CI: 3.15, 3.69) to 4th year (mean 3.70 ± 0.64; 95% CI: 3.48, 3.92). Self-reported confidence in treatment options was significantly different (*p* = 0.02) between class years, with confidence increasing from 2nd year (mean 2.69 ± 0.86; 95% CI: 2.39, 2.99) to 3rd year (mean 3.09 ± 1.01; 95% CI: 2.75, 3.43) to 4th year (mean 3.45 ± 0.83; 95% CI: 3.17, 3.73) (Panel B).

Figure 2 below illustrates self-reported knowledge of epidemiology and genetics (*p* = 0.02), as well as pathophysiology (*p* = 0.031), were significantly different among institutions, with MUSC reporting higher epidemiology and genetics knowledge (mean 3.52 ± 0.65; 95% CI: 3.36, 3.68) and higher treatment knowledge (mean 3.98 ± 9.67; 95% CI: 3.81, 4.15) relative to UNC-CH students’ knowledge of epidemiology and genetics (mean 3.16 ± 0.76; 95% CI: 2.92, 3.40) and treatment (mean 3.65 ± 0.72; 95% CI: 3.42, 3.88); however, adjusting for multiple comparisons, these differences are no longer statistically significant. Between institutions, self-reported confidence in epidemiology and genetics at MUSC (mean 3.48 ± 0.87; 95% CI: 3.26, 3.70) was significantly higher than at UNC-CH (mean 2.86 ± 0.95; 95% CI: 2.55, 3.17).

### 3.4. Self-Reported Knowledge of and Comfort in Treatments of Various Complications

Differences in self-reported knowledge of and comfort in treatment of various complications and conditions (acute pain, acute stroke, pregnancy, acute chest syndrome, and outpatient management) are summarized in Table 2. There is at least one significant difference in students’ knowledge of treatments (*p* < 0.001), with students feeling most knowledgeable about acute pain treatment (mean 3.06 ± 0.98; 95% CI: 2.87, 3.25) and least knowledgeable about pregnancy (mean 2.16 ± 0.96; 95% CI: 1.97, 2.35). Additionally, there is at least one significant difference in students’ comfort of treatments (*p* < 0.001), with students being most comfortable with management of acute pain (mean 3.30 ± 0.91; 95% CI: 3.12, 3.48) and least comfortable with management during pregnancy (mean 2.24 ± 0.92; 95% CI: 2.06, 2.42). There were no significant differences between institutions when adjusting for multiple comparisons (Table S1).

### 3.5. Caring for Patients with Various Complications

**Figure 3:**
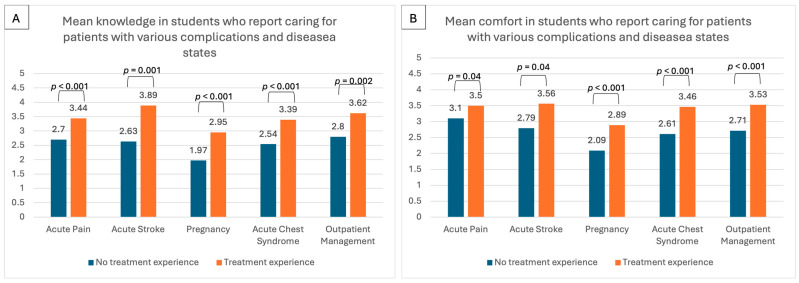
Association of students with treatment experience with various complications compared to those without in terms of knowledge and comfort using Likert scale where 1 = (no knowledge or comfort) to 5 = (extremely knowledgeable or completely comfortable) from SCD-KCS. (**A**) reports self-reported knowledge, while (**B**) reports self-reported comfort. **Note:** *p*-value was determined by the Kruskal-Wallis Test, a non-parametric method for comparing the medians of multiple groups with repeated comparisons adjusted for multiple testing using the Bonferroni correction.

Differences in having treated patients with various disease complications or disease states with self-reported knowledge of and comfort in treatments with various complications are represented in Figure 3. The smallest percentage of individuals reported caring for patients with acute stroke (9/98, 9%). With increasing frequency, 16 (16%) participated in outpatient management, 19 (19%) participated in pregnancy management, 28 (29%) cared for someone with acute chest syndrome, and almost half (48/98, 49%) report caring for someone experiencing acute pain. For each specific condition, students who had treated that condition had greater knowledge and comfort than those who had not (Figure 3). Additional details are provided in Table S2.

### 3.6. Examination of Student’s Knowledge Related to SCD

The overall mean score on the 10 question self-assessment related to SCD was 71% (95% CI: 67.86%, 74.18%). While there were no differences between classes and institutions in the overall 10 question self-assessment, there was a significant difference in at least 1 class year (*p* = 0.026) for questions about managing fever, pain, and stroke. Specifically, when adjusting for multiple comparisons, the MS2 class (mean 48.96%; 95% CI: 40.17%, 57.75%) was significantly lower (*p* = 0.024) than the MS4 class (mean 67.68%; 95% CI: 56.49%, 78.97%).

## 4. Discussion

Our key findings demonstrate more self-reported knowledge and confidence in pre-clinical topics, and overall low knowledge and comfort related to various treatments in SCD. As expected, knowledge and comfort with treatment options increased as individuals progressed through their clinical years. It is not unexpected that students felt most knowledgeable and comfortable about acute pain management, as this is the most common complication in patients with SCD [3]. Finally, providing medical care for patients correlated with increased knowledge and comfort in various treatments related to SCD.

Typically, surveys of providers caring for patients with SCD focus on treatment of pain [7,8,9,11,19,21,22,25,26,27]. One survey of emergency room providers demonstrates high comfort in managing pain from attending providers but low comfort from trainees [25]. In our study, we found that while knowledge and comfort were highest in this category, mean Likert scores were only 3.06 (scale 1–5) and 3.30 (scale 1–5). Interestingly enough, while this is the area where providers often report highest knowledge and comfort [19,22], adherence to guidelines remains poor [27,28]. This suggests that while students are most knowledgeable and comfortable about acute pain management, additional education and support should be provided as they progress through training.

There was no institutional variability in knowledge and comfort/confidence between MUSC and UNC-CH students. These findings are not surprising due to the number of patients with SCD at each of these institutions is similar. Also, despite differences in medical student distributions across institutions many questions excluded individuals who had not yet reached their clinical years. Since caring for patients with SCD positively impacted knowledge of and comfort in management decisions, population size at an institution and access to experts within the field can have a positive impact on learners. In a study conducted by Merz et. al, incorporation of an SCD expert within a general medicine team was demonstrated to improve the care administered to patients with SCD [29]. Involvement of an expert in SCD at the student level could have a similar impact. Considering there are many teaching institutions with a smaller population of patients with SCD, implementation of simulation within curriculum could address this deficit. It has been previously demonstrated that patient simulations can provide useful experiences and increase objective knowledge and confidence relating to SCD [30,31]. These studies show effectiveness on the level of residents and fellows; however, similar approaches could be taken with medical student curriculum.

Previous qualitative work demonstrated residents’ discomfort with taking care of patients with SCD, specifically citing lack of specific education, feeling fear and powerlessness for managing complications, and discomfort caring for patients with SCD [11]. Similar trends were reflected in our study with medical students feeling discomfort with clinical management but had increasing comfort as their experience in caring for those complications increased. Other studies demonstrate that more senior learners within a resident cohort demonstrated higher levels of comfort with acute pain crisis treatment [7]. We echo these findings to further illustrate the importance of foundational learning of SCD at the medical student level.

The lack of knowledge, confidence, and comfort continues beyond residency graduation into practicing physicians. Family medicine providers [12] surveyed about comfort in providing care for patients with SCD reported being uncomfortable providing care in all aspects of SCD management, including routine ambulatory care, managing comorbidities, SCD specific issues, and chronic pain [12]. In this study, providers who had previously cared for patients with SCD expressed more comfort (OR 6.6) caring for these patients in their current practices [12]. Similar data are found through a more recent survey of practitioners as well [22]. Supported by our findings, this discomfort may begin in medical school and continues throughout a practitioner’s career. Another study demonstrated only about 30% of internal medicine and pediatric medicine physicians were comfortable being primary care physicians for those with SCD [21]. Comfort with providing care for patients with SCD was associated with treating a higher number of individuals in their practice. Internists who had provided care to larger volume of patients with SCD in training had more comfort [21,22]. In the SCD-KCS, 49 percent (48/97) of our respondents indicated they plan to enter primary care. Similar to what we demonstrated in our survey, comfort in caring for patients with SCD is rooted in prior experience in both practice and training [21,22]. Our study builds upon the concerns highlighted above and further contributes by enhancing our understanding of medical education surrounding related to this disease process.

In our study, pregnancy management was consistently reported with the least knowledge and comfort in students. This is likely impacted by lack of comfort in practicing providers. More recent obstetric and gynecologic literature demonstrates “somewhat high” or “high SCD knowledge” [22]; however, this contradicts adherence to guidelines demonstrated by another [20]. Reflective of our results, studies report approximately 30% of participants described their training in both medical school and residency as barely adequate or inadequate in caring for patients with SCD [20]. There still exists little information surrounding the experience of obstetrics residents in the care of women with SCD. There are, however, guidelines in the management of pregnant individuals with SCD to guide practitioners, including the British Society of Haematology Guidelines [32], a Delphi consensus published within the last year [33], and American College of Obstetrics and Gynecology consult series on SCD in pregnancy [34]. As emphasized and demonstrated in our study, caring for this population increases comfort across the spectrum of disease [10,35].

To compound these deficits, there are more patients with SCD than hematology providers resulting in students having decreased access to disease experts [18], likely resulting in decreased patient satisfaction and educational opportunities [36]. A study by Kanter, et al. demonstrated patients with SCD report less satisfaction with their experiences with emergency department (ED) doctors and more negative perceived quality of care, which they reported contributed to delays in seeking care [36]. Due to a variety of factors, though, individuals continue to seek care in the ED despite these concerns [37]. Further, adolescent patients with SCD felt they received better care from their primary SCD physician when compared to the ED physician [36]. Another study highlighted that while most centers have a designated adult provider, rarely does that person specialize in adult SCD [38]. Unfortunately, recent literature has demonstrated that the interest in hematology, particularly SCD, among pediatric and adult trainees is decreasing, while the population globally with SCD continues to grow [1,15,16]. Our proposal is that early engagement of students in caring for patients with SCD, alongside access to disease experts during training and proper education, will lead to increased comfort and improved knowledge of management for this often-neglected population.

Finally, we must acknowledge the implicit bias, racism, and stigmatization of these patients and how this underscores the educational emphasis, care, treatment, and research funding surrounding this disease [36,39,40,41,42,43]. It is well known that biases impact willingness to believe patients and adequately treating pain [39,40,41,44,45]. Multiple qualitative studies describe the stigmatization patients of all ages, and their families, experience when seeking care. They are often accused of being “drug seeking” and meet reluctance to be prescribe narcotic medications [44,45,46]. Implicit biases have been reported across the age spectrum as well [39,40,41,47]. SCD is described as an invisible disease, which is thought to be partially impacted by the lack awareness in the healthcare system [39,45]. There is less funding and research for this disease compared with other diseases that do not impact minority population (i.e., cystic fibrosis) [48]. Medical schools across the country are working to increase education surrounding the impact of these injustices on minority patients; however, there are still reports of significant biases toward minority patients [49,50,51,52]. As has been reported elsewhere [39], we demonstrate the role of “patient as teacher” and how it can be particularly impactful for medical students. Our study describes the existing lack of comfort and knowledge related to SCD. We hope to highlight the experiences of individuals living with this disease and improve care throughout their lives to lessen these difficulties.

Our study has several limitations. First, this was designed as a pilot study to determine the adequacy of a survey within two institutions to describe student knowledge, confidence, and comfort with caring for individuals with SCD. In as much, the SCD-KCS was not a validated instrument; however, there are no validated tools developed for such indication. In future studies, we are planning for additional analysis as well to further support validation of this instrument and analysis of its reliability and internal consistency of the SCD-KCS. We looked only at two similarly sized public medical schools in the Southeastern United States with large populations of patients with SCD. These are likely generalizable to other schools with similar patient demographics nationwide, but we cannot be certain. Next steps will include a revised version of the SCD-KCS with wider distribution to additional institutions to improve generalizability. We did not assess curriculum surrounding SCD, which could provide insight into responses as well. Though with any additions, this must be balanced by the burden added to those completing the survey. We also were not able to have students specify in what setting, when, and how many patients with SCD they provided care. We see this as an area that could have provided significant insight into the data in hindsight. We did notice a distribution of students skewed toward more MS2 at MUSC and more MS4 at UNC-CH, which likely affected the distribution of some responses as well. Overall, due to the findings and scope of this study as it relates to the topic of SCD education in medical education, we feel it adds significant value to the literature.

## 5. Conclusions

Through survey-based methods, we determined medical students’ knowledge, confidence, and comfort related to caring for patients with SCD. We found the following key points: (1) preclinical topics of pathophysiology, genetics, and epidemiology were associated with higher self-reported knowledge and confidence compared with treatment options, (2) overall knowledge and comfort in treating specific complications were low, and (3) experience providing care for individuals with certain complications led to significant increases in knowledge and comfort across all areas. This work shows the need to improve education surrounding SCD at the medical student’s level, as this may directly impact long-term patient outcomes. We strive to have this impact through two key actions: development of supplemental medical school curricula focused on SCD, and investigation of how this influences individuals’ career choices and patient outcomes.

## Figures and Tables

**Table 1:** Demographic information regarding students who participated in the SCD-KCS presented as combined overall data, MUSC specific data, and UNC specific data followed by the *p*-values.

	Overall	MUSC	UNC	*p*-Value
Number of Subjects	98	61	37	
**Year**				<0.001
MS2	32 (33%)	31 (51%)	1 (3%)	
MS3	33 (34%)	21 (34%)	12 (32%)	
MS4	33 (34%)	9 (15%)	25 (68%)	
**Gender**				1.000
Female	67 (68%)	42 (69%)	25 (68%)	
Male	29 (30%)	18 (30%)	11 (30%)	
Other	2 (2%)	1 (2%)	1 (3%)	
**Race**				0.695
White	57 (61%)	37 (64%)	20 (56%)	
Black/AA	12 (13%)	8 (14%)	4 (11%)	
White/AA	2 (2%)	1 (2%)	1 (3%)	
Asian	22 (23%)	11 (19%)	11 (31%)	
Other	1 (1%)	1 (2%)	0 (0%)	
**Ethnicity**				0.471
Latinx/Hispanic	8 (8%)	4 (7%)	4 (11%)	
Non-Hispanic	90 (92%)	57 (93%)	33 (89%)	
**Exposure to SCD**				
Training/Workshop = Yes (%)	23 (24%)	20 (33%)	3 (8%)	0.006
Personal Experience = Yes (%)	5 (5.%)	4 (7%)	1 (3%)	0.647
Education Exposure, Readings, Lectures = Yes (%)	97 (99%)	60 (98%)	37 (100%)	1.000
Media = Yes (%)	34 (35%)	20 (33%)	14 (38%)	0.665
Other = Yes (%)	5 (5%)	3 (5%)	2 (5%)	1.000
**Career Goal**				0.652
Primary Care	48 (50%)	28 (47%)	20 (54%)	
Subspecialty Care	20 (21%)	12 (20%)	8 (22%)	
Surgical	29 (30%)	20 (33%)	9 (24%)	

**Note**: *p*-value was determined using Fisher’s Exact Test to compare differences in categories between institutions. For analysis, education exposure, readings, and lectures were combined into one variable, and responding yes to any counted as 1 answer. This does not include those who had exposure to patients in clinical capacity. Due to rounding to the whole number, some percentages will be >100%.

**Table 2:** Mean results from knowledge and comfort in treatment of various complications and those with SCD using Likert Scale with 1 = (no knowledge or comfort) and 5 = (extremely knowledgeable or completely comfortable) from SCD-KCS.

	Overall: Mean (SD)	*p*-Value
**Knowledge of Treatment**		
Acute Pain	3.06 (0.98)	<0.001
Acute Stroke	2.74 (1.07)	
Pregnancy	2.16 (0.96)	
Acute Chest Syndrome	2.79 (1.06)	
Outpatient Management	2.94 (1.00)	
**Comfort in Treatment**		
Acute Pain	3.30 (0.91)	<0.001
Acute Stroke	2.86 (1.01)	
Pregnancy	2.24 (0.92)	
Acute Chest Syndrome	2.86 (0.99)	
Outpatient Management	2.84 (0.98)	

**Note:** *p*-value was determined by Friedman’s Test for Ordinal Measures, a non-parametric method for comparing more than two related groups based on ordinal measures.

## Data Availability

The data supporting the findings in this study are available from the corresponding author upon reasonable request.

## Supplementary Materials

The following supporting information can be downloaded at: https://www.mdpi.com/article/10.3390/healthcare13151909/s1.

## Abbreviations

The following abbreviations are used in this manuscript:

SCD	Sickle cell disease
RBC	Red blood cell
MUSC	Medical University of South Carolina
UNC-CH	University of North Carolina, Chapel Hill
REDCap	Research Electronic Data Capture
MS	Medical students
ED	Emergency department
