# Supplementary material for: Knowledge, Confidence, and Comfort Regarding Sickle Cell Disease Among Medical Students: A Pilot Study in Two Universities
Source: Healthcare (Basel). 2025 Aug 5;13(15):1909. doi: 10.3390/healthcare13151909 (PMC12346322; doi:10.3390/healthcare13151909)
Supplement: Supplementary file 1 [file healthcare-13-01909-s001.zip › healthcare-3648499-supplementary.pdf]

Supplemental Materials

How Exposure to Patients with Sickle Cell Disease Changed Knowledge, Confidence, and Comfort with Care: A Pilot Survey of Medical Students

Authors: Christina M. Abrams, MD[1], DeAsia Witherspoon[2], Everette Keller, MS[3], Andrew J Picca, DO[1], MAEd, Maria Boucher, MD[4]

[1]Department of Pediatrics, Division of Hematology/Oncology, Medical University of South Carolina, Charleston, SC, C.M.A. abramsch@musc.edu

[2]College of Medicine, Medical University of South Carolina, Charleston, SC, withedea@musc.edu

[3]Department of Public Health Sciences, College of Medicine, Medical University of South Carolina, Charleston, SC, kellerev@musc.edu

[4]Department of Pediatrics, Division of Hematology/Oncology, University of North Carolina, Chapel Hill, NC, Maria_Boucher@med.unc.edu

****Correspondence**: Department of Pediatrics, Division of Hematology/Oncology, 125 Doughty Street, Suite 520, Charleston, SC 29403, abramsch@musc.edu

Contents:

Supplement S1:	Distribution email provided to students including informed consent, privacy, and rights of participation.	Page 2
Supplement S2:	Sickle Cell Knowledge and Comfort Survey distributed to students via RedCap.	Page 3
Table S1:	Knowledge and comfort in treatment of various complications and care those with SCD.	Page 8
Table S2:	Association of individuals reporting treatment experience with various complications compared to those without in terms of knowledge and comfort.	Page 9

<u>Supplemental Material S1: Email distribution to students at MUSC and UNC-CH for survey participation.</u>

This survey included details of participation, link to the survey, and statements about informed consent, privacy, rights of participants, and instructions as below.

<u>Subject of email:</u> Survey participation for medical student knowledge of Sickle Cell Disease

We are trying to better understand what education and exposure medical students are receiving in regards to learning about and treating patients with sickle cell disease during their medical training. There are approximately 100,000 Americans living with sickle cell disease in the United States, and the likelihood of encountering a patient with sickle cell disease is high during training and practice. This study aims to assess and describe medical students' comfort and knowledge for caring for those with sickle cell disease based on the teachings and experiences they had during medical school.

We ask you take the time to complete this survey by [2 weeks after distribution].

Thank you,

DeAsia Witherspoon

[SURVEY LINK]

Principal Investigators:

DeAsia Witherspoon, MS2
withedea@musc.edu

Tina Abrams, MD
abramsch@musc.edu

Maria Boucher, MD
maria_boucher@med.unc.edu

INFORMED CONSENT: We have obtained permission to distribute this survey to medical students from the Institutional Review Board (IRB) of the University of North Carolina (UNC) School of Medicine and the Medical University of South Carolina (MUSC) School of Medicine. By filling out the survey you are consenting to participation in this research project.

PRIVACY: Your privacy will be maintained in all published and written data resulting from the survey. We will not use your email address for any non-research purposes without your consent. We will not share institution level data, only aggregate, de-identified data at scientific forums.

RIGHTS OF PARTICIPANTS: Your contribution is voluntary, and you have the right to discontinue the survey at any time. You have the right to refuse to answer specific questions. Not participating will not affect your standing within your respective medical school.

INSTRUCTIONS: The survey was designed to be easy to complete. We estimate this should take only about 7 minutes to complete.

If you have any questions or concerns, please contact:

DeAsia Witherspoon
withedea@musc.edu

Or

MUSC IRB:
(843)-792-4148

UNC IRB:
irb_subjects@unc.edu
(919) 966-3113

Supplemental Material S2: Sickle Cell Knowledge and Comfort Survey

Survey was distributed by email using RedCap platform for them to complete online as described in the manuscript.

Part I. Demographic Information

At what institution are currently attending medical school? ____________

What year in medical school are you?

- 2nd year
- 3rd year
- 4th year

Please mark all that apply:

- Female
- Male
- Transgender or Gender Nonconforming
- Different Identity

Please mark all that apply:

- Latinx/Hispanic
- Black/African American
- White
- Asian
- Native American/Alaskan or Pacific Islander
- Other ________________

What previous exposure, if any, do you have regarding Sickle Cell Disease? Please mark all that apply:

- Training/Workshops
- Personal Experience (self, family, friend)
- Educational Experience (patient exposure)
- Readings (textbooks, academic journal articles)
- Lectures (in college, medical school)
- Media (podcasts, educational videos, interviews, etc.)
- Other Sources _____________________
- I do not have any previous exposure to Sickle Cell Disease

What are your career goals after medical school?

- Primary Care (family medicine, pediatrics, internal medicine, psychiatry, ob-gyn, etc)
- Subspecialty Care (dermatology, pathology, emergency medicine, radiology, etc)
- Surgical Subspecialty (general surgery, orthopedics, urology, neurosurgery, etc)
- Other

Part II. Self-Assessment

During my hematology block, we discussed sickle cell disease?

- Yes
- No
- I don't remember.

During my preclinical years, my medical school addressed the following areas regarding sickle cell disease (select all that may apply):

- Pathophysiology
- Epidemiology
- Genetics
- Treatment/Pain Management options
- Complications

During my clinical years, I cared for those with sickle cell disease with the following diagnoses (select all that may apply)

- o Acute pain
- o Acute stroke
- o Pregnancy
- o Blood clot
- o Acute chest syndrome
- o Fever
- o Outpatient Care
- o I did not care for a single patient who carried a diagnosis of sickle cell disease during my clinical years.
- o I have not started my clinical rotations yet.

Part III

Please rate your level of <u>KNOWLEDGE</u> on the following…	No Knowledge 0	1	2	3	Extremely Knowledgeable 4
The genetics and epidemiology of Sickle Cell Disease	0	1	2	3	4
The pathophysiology of Sickle Cell Disease	0	1	2	3	4
The treatment of Sickle Cell Disease	0	1	2	3	4

How would you rate your knowledge of treatment options for patients with sickle cell disease in the following areas?	No knowledge 0	1	2	3	Extremely knowledgeable 4
Vaso-occlusive pain	0	1	2	3	4
Acute chest syndrome	0	1	2	3	4
Stroke	0	1	2	3	4
Pregnancy	0	1	2	3	4
Outpatient management	0	1	2	3	4

Please rate how much <u>CONFIDENCE</u> do you have in your ability to…	No Confidence 0	1	2	3	Complete Confidence 4
Describe the genetics and epidemiology of Sickle Cell Disease.	0	1	2	3	4
Describe the pathophysiology of Sickle Cell Disease.	0	1	2	3	4
Describe the treatment of Sickle Cell Disease.	0	1	2	3	4

What is your comfort with the following topics regarding sickle cell disease?	No comfort 0	1	2	3	Completely comfortable 4
Vaso-occlusive pain	0	1	2	3	4
Acute chest syndrome	0	1	2	3	4
Stroke	0	1	2	3	4
Pregnancy	0	1	2	3	4
Outpatient management	0	1	2	3	4
Pathophysiology of sickle cell disease	0	1	2	3	4
Drug therapy for managing sickle cell disease	0	1	2	3	4

Objective-Assessment
Please choose the best answer.

1. What is the cause of SCD?
    a. Idiopathic
    b. Acquired
    c. Hereditary
    d. I don't know.
2. Which of the following is the mutation responsible for causing Sickle Cell Disease?
    a. Glu → Val
    b. Val → Glu
    c. Asp → Glu
    d. Glu → Asp
3. Which of the following is NOT a recommended treatment for pediatric patients with Sickle Cell Disease?
    a. Hydroxyurea
    b. Blood transfusion
    c. Vincristine
    d. Bone marrow transplant
4. Sickle cell disease is caused by…
    a. Hemoglobin polymerization within the red blood cell
    b. Deoxygenated hemoglobin
    c. Hemolysis
    d. Dehydration
5. Which is a preventive measure that can be taken for SCD?
    a. Pre-marital screening
    b. Medical advice
    c. Blood transfusion
    d. I don't know.
6. Which of the following types of SCD are typically considered more severe?
    a. HbSS and HbSC
    b. HbSS and HbSB^{+}Thal
    c. HbSS and HbSB^{0}Thal
    d. HbSC and HbSB^{+}Thal
7. Patients with sickle cell at increased risk for infection with which bacteria?
    a. Staphylococcus aureus
    b. Streptococcus pneumoniae
    c. Escherichia coli
    d. Vibrio cholerae
8. 2-year-old male with SCD presents to the ER with a fever of 103°F at home. He has a runny nose and congestion. He is well appearing, and his vital signs are stable. He currently has no pain. What work-up should be done for him?
    a. Nothing, send home with supportive care
    b. Blood culture, labs, and a dose of Ceftriaxone
    c. Labs, IV fluids, and IV pain medicine
    d. Labs, including type and screen, blood transfusion
9. 10-year-old female with HbSS presents with right upper extremity pain, that is typical for her SCD. They tried their home pain medication without any signs of improvement. How would you treat this patient?
    a. IV fluids and oral pain medicine
    b. Oxygen
    c. IV pain medicine and Toradol
    d. Ultrasound to rule out clot
10. 5-year-old male with HbSS with a history of abnormal TCD (stroke screen) presents to the ED with left sided weakness. What is the next best step?
    a. Emergent red blood cell exchange transfusion
    b. IV pain medicine

c. Tell them to follow up with their pediatrician tomorrow
d. Recombinant tPa

Table S1 – Knowledge and comfort in treatment of various complications and care those with SCD using Likert Scale with 1 = (no knowledge or comfort) and 5 = (extremely knowledgeable or completely comfortable).

	Institution		p-value#
	MUSC	UNCCH	
Knowledge of Treatment			
Acute Pain	3.00 (1.03)	3.16 (0.90)	1.000
Acute Stroke	2.74 (1.14)	2.76 (0.95)	1.000
Pregnancy	2.33 (1.03)	1.89 (0.77)	0.216
Acute Chest Syndrome	2.72 (1.11)	2.89 (0.97)	1.000
Outpatient Management	2.93 (0.98)	2.95 (1.05)	1.000
Comfort in Treatment			
Acute Pain	3.31 (0.96)	3.27 (0.84)	1.000
Acute Stroke	2.90 (1.00)	2.78 (1.03)	1.000
Pregnancy	2.39 (0.92)	2.00 (0.88)	0.190
Acute Chest Syndrome	2.82 (1.01)	2.92 (0.98)	1.000
Outpatient Management	2.92 (0.94)	2.69 (1.04)	1.000

# p-value was determined by the Kruskal Wallis Test, a non-parametric method for comparing the medians of multiple groups with repeated comparisons adjusted for multiple testing using the Bonferroni correction.

Table S2 – Association of individuals reporting treatment experience with various complications compared to those without in terms of knowledge and comfort using Likert scale where 1 = (no knowledge or comfort) to 5 = (extremely knowledgeable or completely comfortable.

	Number of Individuals Reporting treatment experience: n (%)	Knowledge: mean (sd)		p-value#	Comfort: mean (sd)		p-value#
		No Treatment experience	Treatment experience		No Treatment experience	Treatment experience	
Acute Pain	48 (49%)	2.70 (1.02)	3.44 (0.80)	< 0.001	3.10 (0.95)	3.50 (0.83)	0.036
Acute Stroke	9 (9.2%)	2.63 (1.02)	3.89 (0.93)	0.001	2.79 (0.98)	3.56 (1.01)	0.043
Pregnancy	19 (19.4%)	1.97 (0.88)	2.95 (0.91)	< 0.001	2.09 (0.89)	2.89 (0.74)	< 0.001
Acute Chest Syndrome	28 (28.6%)	2.54 (1.03)	3.39 (0.88)	< 0.001	2.61 (0.92)	3.46 (0.92)	< 0.001
Outpatient Management	16 (16.3%)	2.80 (1.00)	3.62 (0.72)	0.002	2.71 (0.97)	3.53 (0.64)	0.001

Note: # p-value was determined by the Kruskal Wallis Test, a non-parametric method for comparing the medians of multiple groups with repeated comparisons adjusted for multiple testing using the Bonferroni correction.
